# 

**Published:** 1889-05-25

**Authors:** 


					Jo. iffl, Vol. IVI,
May 2btii, UHtfy,
. [Registered at the General Post Office as a .Newspaper ]
Monthly Parts, 6d.; post-free 7?d.
Ditto per Annum, 7s. 6d., post free.

				

